# Prognostic Model to Predict Overall Survival for Metastatic Non-Small Cell Lung Cancer Patients Treated With Chemotherapy Combined With Concurrent Radiation Therapy to the Primary Tumor: Analysis From Two Prospective Studies

**DOI:** 10.3389/fonc.2021.625688

**Published:** 2021-02-25

**Authors:** Ling-Feng Liu, Qing-Song Li, Yin-Xiang Hu, Wen-Gang Yang, Xia-Xia Chen, Zhu Ma, Wei-Wei OuYang, Yi-Chao Geng, Cheng Hu, Sheng-Fa Su, Bing Lu

**Affiliations:** ^1^Department of Oncology, Affiliated Hospital of Guizhou Medical University, Guiyang, China; ^2^Department of Oncology, Guizhou Cancer Hospital, Guiyang, China

**Keywords:** prognostic model, overall survival, metastasis, non-small cell lung cancer, chemoradiotherapy

## Abstract

**Purpose:**

The role of radiotherapy, in addition to chemotherapy, has not been thoroughly determined in metastatic non-small cell lung cancer (NSCLC). The purpose of the study was to investigate the prognostic factors and to establish a model for the prediction of overall survival (OS) in metastatic NSCLC patients who received chemotherapy combined with the radiation therapy to the primary tumor.

**Methods:**

The study retrospectively reviewed 243 patients with metastatic NSCLC in two prospective studies. A prognostic model was established based on the results of the Cox regression analysis.

**Results:**

Multivariate analysis showed that being male, Karnofsky Performance Status score < 80, the number of chemotherapy cycles <4, hemoglobin level ≤120 g/L, the count of neutrophils greater than 5.8 ×10^9^/L, and the count of platelets greater than 220 ×10^9^/L independently predicted worse OS. According to the number of risk factors, patients were further divided into one of three risk groups: those having ≤ 2 risk factors were scored as the low-risk group, those having 3 risk factors were scored as the moderate-risk group, and those having ≥ 4 risk factors were scored as the high-risk group. In the low-risk group, 1-year OS is 67.7%, 2-year OS is 32.1%, and 3-year OS is 19.3%; in the moderate-risk group, 1-year OS is 59.6%, 2-year OS is 18.0%, and 3-year OS is 7.9%; the corresponding OS rates for the high-risk group were 26.2%, 7.9%, and 0% (P<0.001) respectively.

**Conclusion:**

Metastatic NSCLC patients treated with chemotherapy in combination with thoracic radiation may be classified as low-risk, moderate-risk, or high-risk group using six independent prognostic factors. This prognostic model may help design the study and develop the plans of individualized treatment.

## Introduction

More than half of non-small cell lung cancer (NSCLC) patients have distant metastases at the time of initial diagnosis (1). Drug therapy is the main treatment for metastatic NSCLC. Molecular targeted therapy is recommended for metastatic NSCLC patients if sensitive gene aberrations are detected (2). Molecular targeted therapy has less toxicity and higher efficacy in comparison with conventional chemotherapy (3, 4). However, only approximately 30% of the patients may have positive responses from the molecular targeted therapy (5, 6), and therefore about 70% of patients require other systemic therapy (7, 8).

However, antibodies to the programmed death protein 1 (PD-1), such as the monotherapy drug pembrolizumab, can be utilized as a first-line treatment for the metastatic NSCLC patients, without sensitizing the anaplastic lymphoma kinase (ALK) or epidermal growth factor receptor (EGFR) changes with the programmed death ligand 1 (PD-L1) tumor proportion score (TPS) of 1% or higher (9). However, the cost of pembrolizumab is high, and many patients cannot afford pembrolizumab treatment. For NSCLC patients, pembrolizumab may not be considered cost-effective in treatment (10, 11). Thus, platinum-based doublet chemotherapy is the most extensively utilized modality in treating metastatic NSCLC patients without sensitive gene aberrations (8).

Platinum-based doublet chemotherapy generally produces about 30% response rates, with a median overall survival of 8 to 10 months, and similar efficacy with different chemotherapy regimens (12, 13). For patients with metastatic NSCLC, oncologists focused more on systemic therapy to control the metastatic lesions than local treatment to control the primary tumor. However, nearly 50% of patients with metastatic diseases have a local recurrence at the initial site of involvement, and local control and status of the primary tumor are associated with OS (14, 15). Our previous prospective studies and other retrospective studies showed that chemotherapy with concurrent thoracic radiation to the primary tumor produces favorable survival outcomes with acceptable toxicity (16–18). Yen et al. also demonstrated that the survival benefits of combining thoracic RT (45 Gy at least) and EGFR tyrosine kinase inhibitor (TKI) in metastatic NSCLC patients with sensitizing EGFR alterations (19). At present, increasing evidence shows that local treatment to all metastatic lesions can improve survival outcomes in patients with oligometastatic NSCLC (20–22). These publications suggested that some patients with metastatic diseases could benefit from the thoracic radiation to the primary tumor with system treatment (16–19). However, routine use of concurrent thoracic chemoradiation is not recommended for patients with metastatic NSCLC (23). A well-defined risk scoring system is essential to identify metastatic NSCLC patients who may benefit from chemotherapy with concurrent thoracic radiation to the primary tumor. The aim of this study is to determine the prognostic factors and to establish a model for the prediction of overall survival (OS) in metastatic NSCLC patients who received chemotherapy combined with radiation therapy to the primary tumor.

Clinical characteristics, such as gender, pathological type, weight loss, KPS, age, and metastatic status, are important prognostic factors of metastatic NSCLC. Furthermore, laboratory parameters, such as white blood cells, hemoglobin, platelets, fibrinogen, albumin, and lactate dehydrogenase, are also related to the prognosis of NSCLC (16, 24–28). These laboratory parameters are the routine testing items of metastatic NSCLC, which can be obtained conveniently and economically. Due to the uncertainties regarding concurrent thoracic radiotherapy in combination with chemotherapy, we hope to develop a prognostic model that is convenient for clinical application to estimate the overall survival outcome (23).

In our present study, we have developed a prognostic model on the basis of the parameters of pretreatment laboratory and clinical characteristics of metastatic NSCLC patients from two prospective studies (17, 18). Our purpose was to stratify patients into different risk groups and to identify a subgroup that may benefit from thoracic radiotherapy with concurrent chemotherapy.

## Material and Methods

### Patients Selection

We retrospectively analyzed 243 eligible patients with metastatic NSCLC who were treated with chemotherapy and concurrent radiation to the primary tumor in two prospective studies (17, 18). The selection criteria were as follows: (1) histologically or cytology confirmed NSCLC; (2) newly diagnosed metastatic disease limited to ≤3 organs; (3) did not receive targeted therapy or immunotherapy during lifetime; (4) aged 18–75 years; (5) a Karnofsky Performance Status (KPS) score ≥70; (6) received at least two chemotherapy cycles and a thoracic radiation dose of at least 40 Gy in 2-Gy fractions; (7) received three-dimensional conformal radiation therapy [3DCRT] or intensity modulated radiation therapy [IMRT]; (8) had complete clinicopathologic and follow-up information; (9) had pretreatment records of blood routine, blood biochemistry, and coagulation function test within 1 week before treatment. This study was reviewed by the ethical review boards in China (Ethics Committee of Guizhou Cancer Hospital, Guiyang, China), and informed consents were obtained from all patients.

### Clinical and Laboratory Data Collection

Clinicopathologic information included sex, age, KPS score, tumor histology, N stage, T stage, metastatic status at diagnosis, size of primary tumor, tumor size, and survival outcomes (dead or alive). Laboratory testing parameters included white blood cell (WBC) count, hemoglobin (Hb) level, platelet (PLT) count, neutrophil absolute value, lymphocyte absolute value, Neutrophil-to-Lymphocyte Ratio (NLR), albumin, serum calcium level, lactate dehydrogenase (LDH), alkaline phosphatase (ALP) level, plasma D-dimer, and fibrinogen.

### Treatment Methods

All patients received the following first-line therapy: docetaxel plus cisplatin given every 21–28 days concurrent with thoracic radiation therapy. No induction chemotherapy was given before radiation. After the completion of thoracic radiotherapy, patients who were in remission or had stable disease continued chemotherapy for a total of 4–6 cycles. No maintenance therapy was given. Modern techniques (3D-CRT or IMRT) were utilized to deliver at least 40 Gy thoracic radiation dose (2 Gy per fraction) to all enrolled cases. It is noted that radiotherapy and chemotherapy were given concurrently, and that radiotherapy commenced within one week after the administration of the first course of chemotherapy. Details of the radiation therapy protocol were reported previously (17, 18).

### Follow-up and Statistical Analyses

The overall survival (OS) time was measured from the starting date of treatment to the date of death or the last follow-up. The Statistical Package for Social Sciences, version 15.0 (SPSS, Chicago, IL, USA) was used for statistical analysis. Receiver operating characteristic (ROC) curves with binary variable of OS longer or shorter than 10.0 months and Youden’s index were used to determine the best cut-off value for baseline values of continuous variables, such as white blood cell count, platelet count, hemoglobin level, etc., as a prognostic factor. The Kaplan-Meier method was used to calculate the OS, and the curves were compared with log-rank test results. Multivariate Cox regression analysis was utilized to test independent significant prognostic factors for OS. All factors with *P* value ≤0.10 in univariate analysis were further tested in the multivariate analysis. We developed a prognostic model to predict the survival of NSCLC patients based on the results of the Cox regression analysis. Harrell’s test was used to validate the model. All statistical tests were 2-sided, and *P* values < 0.05 were considered statistically significant.

## Results

### Patient Characteristics

A total of 243 eligible patients were included in this study, including 154 male and 89 female patients, aged 26–75 (median: 58) years. The most common site of metastatic disease at diagnosis was the bone (51.4% of patients); 90 (37%) had brain metastases and 78 (32%) patients had lung metastases. One hundred and five (43.2%) patients had a single metastasis. The clinical characteristics of the 243 patients are listed in **Table 1**.

**Table 1:** Characteristics of 243 patients with metastatic NSCLC.

Characteristic	No. (%)
Gender	
Male	154 (63.4)
Female	89 (36.6)
Age	
Median (range)	58 (26–75)
<60 years	172
≥60 years	71
Tumor histology	
Squamous	78 (32.1
Nonsquamous	165 (67.9)
History of smoking	
Yes	128 (52.7)
No	115 (47.3)
weight loss >5% in the last 6 months	
Yes	52 (21.4)
No	191 (78.6)
KPS	
<80	21 (8.6)
≥80	222 (91.4)
T stage	
T_1-2_	69 (28.4)
T_3-4_	174 (71.6)
N stage	
N_0-1_	29 (11.9)
N_2-3_	214 (88.1)
No. of metastatic organs	
1	105(43.2)
2	77 (31.7)
3	61 (25.1)
Total number of metastases	
≥5	71 (29.3)
<5	172 (70.7)
Brain Metastasis	
Yes	90 (37)
No	153 (63)
Bone Metastasis	
Yes	125 (51.4)
No	118 (48.6)
Liver Metastasis	
Yes	24 (9.9
No	219 (90.1)
Contralateral lung metastasis	
Yes	78 (32.1)
No	165 (67.9)
Radiotherapy technology	
3DCRT	85 (34.9)
IMRT	158 (65.1)
Radiation to metastases	
Yes	157 (64.6)
No	86 (35.4)
No. of chemotherapy cycle	
<4	122 (50.2)
≥4	121 (49.8)
D-Dimer (mg/L)	
<0.5	118 (48.6)
≥0.5	125 (51.4)
Radiotherapy dose, Av ± SD	56.4 ± 23.11(Gy)
Fibrinogen, Av ± SD	4.07 ± 1.18 (g/L)
WBC count, Av ± SD	7.23 ± 2.53 (10^9^/L)
Hb level, Av ± SD	130.45 ± 17.80 (g/L)
PLT count, Av ± SD	250.0 ± 99.61 (10^9^/L)
Neutrophil absolute value, Av ± SD	4.96 ± 2.28 (10^9^/L)
Lymphocyte absolute value, Av ± SD	1.35 ± 0.569 (10^9^/L)
Neutrophil-to-lymphocyte ratio, Av ± SD	4.39 ± 4.34
Serum calcium level, Av ± SD	2.30 ± 0.76 (nmol/L)
Albumin, Av ± SD	41.0 ± 3.99 (g/L)
Lactate dehydrogenase, Av ± SD	228.63 ± 117.0 (U/L)
ALP level, Av ± SD	123.87 ± 106.28 (U/L)
Maximum diameter of primary tumor, Av ± SD (range)	4.78 ± 2.30 (cm)

Av, average; SD, standard deviation.

### Survival Outcomes and Prognostic Factors

The follow-up periods ranged from 2.0 to 64.0 months, with a median follow-up period of 14.0 months. The median OS time for all patients was 13.0 months (95% confidence interval [CI]: 11.7–14.3), and the OS rates at 1, 2, and 3 years were 55.2%, 17.8%, and 11.0% respectively. Univariate analysis showed that sex, KPS score, T status, number of metastatic organs, brain metastasis, number of chemotherapy cycles, plasma D-dimer, fibrinogen level, WBC count, Hb level, PLT count, neutrophil count, NLR, serum albumin level, and ALP level were associated with OS significantly (**Table 2**). Multivariate analysis showed that being male, KPS score < 80, the number of chemotherapy cycles <4, Hb level ≤ 120g/L, neutrophil count >5.8 ×10^9^/L, and PLT count >220 ×10^9^/L had a negative effect on OS, as shown in **Table 3**.

**Table 2:** Univariate analysis of factors potentially associated with overall survival outcomes.

Variable	No.	Median OS	Overall survival rate (%)			*P* Value
			1 y	2 y	3 y	
Gender						0.022
Male	154	12.0	50.0	17.9	7.5	
Female	89	15.0	61.8	22.4	18.0	
Age (years)						0.334
<60	172	13.7	57.6	19.7	10.5	
≥60	71	11.6	46.5	18.9	12.9	
Tumor histology						0.388
Squamous	78	13.0	51.3	16.3	4.1	
Nonsquamous	165	13.4	55.8	20.8	14.8	
History of smoking						0.078
Yes	128	12.0	46.9	18.5	8.0	
No	115	15.0	62.6	20.8	15.3	
weight loss >5% in the last 6 months						0.339
Yes	52	15.0	67.2	24.4	8.7	
No	191	12.1	50.8	18.3	11.8	
KPS						0.002
< 80	21	7.0	28.6	4.8	0	
≥ 80	222	13.6	56.8	21.0	12.5	
T stage						0.009
T_1-2_	69	14.7	44.9	22.6	14.6	
T_3-4_	174	11.0	58.0	11.8	0	
N stage						0.880
N_0-1_	29	13.0	58.6	10.3	0	
N_2-3_	214	13.0	53.7	20.9	12.3	
No. of metastatic organs						0.018
≤2	182	13.7	57.7	23.0	13.5	
>2	61	11.3	44.3	9.1	4.6	
Total number of metastases						0.000
< 5	172	60.5	24.3	14.5	15.2	
≥ 5	71	39.4	5.6	4.2	11.0	
Brain metastasis						0.000
Yes	90	11.0	44.4	7.6	2.5	
No	153	15.2	59.5	26.7	16.6	
Bone metastasis						0.054
Yes	125	12.0	48.8	14.1	9.7	
No	118	15.0	60.2	25.4	12.8	
Liver metastasis						0.669
Yes	24	11.6	41.7	16.7	11.1	
No	219	13.6	55.7	19.9	11.4	
Contralateral lung metastasis						0.408
Yes	78	12.0	50.0	19.7	7.8	
No	168	13.4	56.4	19.4	12.9	
Radiotherapy doseGy						0.001
<60	112	42.0	12.6	8.7	11.0	
≥60	131	64.9	24.0	13.9	16.0	
Radiotherapy technology						0.783
3DCRT	85	54.1	17.3	3.8	14.0	
IMRT	158	54.4	19.2	14.4	12.7	
Radiation to metastases						0.582
Yes	157	54.8	18.3	10.6	13.0	
No	86	53.5	19.6	12.7	13.4	
No. of chemotherapy cycle						0.003
<4	122	10.0	40.2	14.9	10.2	
≥4	121	16.0	68.6	23.4	13.4	
D-Dimer (mg/L)						0.040
<0.5	118	16.0	61.0	21.5	13.2	
≥0.5	125	12.0	48.0	18.0	9.5	
Fibrinogen, g/L						0.000
≤4.50	175	15.0	62.3	23.1	14.0	
>4.50	68	9.0	33.8	10.1	4.0	
WBC count, ×10^9^/L						0.030
≤7.5	165	15.0	63.0	21.5	11.2	
>7.5	78	9.1	35.9	15.4	10.8	
Hb level, g/L						0.000
≤120	80	9.7	37.5	11.3	6.5	
>120	163	16.0	62.6	22.8	13.6	
PLT count, ×10^9^/L						0.001
≤220	112	16.0	67.0	25.7	15.9	
>220	131	11.0	43.5	14.2	7.3	
Neutrophil absolute value, ×10^9^/L						0.000
≤5.8	176	15.0	63.1	22.4	12.6	
>5.8	67	9.0	31.3	11.9	6.8	
Lymphocyte absolute value, ×10^9^/L						0.094
≤0.93	43	10.0	34.9	12.4	9.3	
>0.93	200	14.0	58.5	21.1	11.6	
Neutrophil-to-lymphocyte ratio						0.001
≤4.30	149	15.2	62.4	25.6	15.2	
>4.30	94	11.0	41.5	10.1	5.4	
Serum calcium level, nmol/L						0.131
≤2.35	150	12.0	50.0	18.8	9.7	
>2.35	93	15.0	61.2	20.6	11.9	
Albumin, g/L						0.048
≤40	64	9.0	40.6	16.8	8.7	
>40	179	14.0	59.2	20.6	12.2	
LDH, U/L						0.062
≤165	61	16.7	67.2	27.7	12.0	
>165	182	12.0	50.0	16.8	11.0	
ALP level, U/L						0.009
≤100	129	15.4	62.0	26.5	15.7	
>100	114	11.6	45.6	11.6	7.4	
Maximum diameter of primary tumor						0.130
≤3.65 cm	91	12.0	46.2	17.3	9.7	
>3.65 cm	152	14.7	59.2	20.9	12.3	

**Table 3:** Multivariate analysis of factors for the prediction of overall survival.

	β	HR	95.0% CI		*P*
			Lower	Upper	
Gender, (male vs. female)	0.589	1.802	1.102	2.947	0.019
Neutrophil absolute value, (>5.8 vs. ≤5.8 ×10^9^/L)	0.518	1.678	1.053	2.676	0.030
KPS, (<80 vs. ≥80)	0.591	1.806	1.086	2.737	0.023
Hb level, (<120 vs. ≥120 g/L)	0.475	1.608	1.138	2.271	0.007
PLT count, (>220 vs. ≤ 220 ×10^9^/L)	0.470	1.601	1.173	2.185	0.003
No. of chemotherapy cycles, (<4 vs. ≥4)	0.339	1.404	1.014	1.943	0.041
Radiotherapy dose, (<60 vs. ≥60Gy)	0.290	1.336	0.957	1.865	0.089
Total number of metastases, (≥5 vs.<5)	0.318	1.374	0.933	2.025	0.108
WBC count, (≤ 7.6 vs. >7.6 ×10^9^/L)	0.218	1.243	0.841	1.839	0.276
No. of metastatic organs, (≥2 vs. <2)	0.046	1.047	0.697	1.573	0.826
History of smoking, (No vs. Yes)	0.113	1.120	0.714	1.756	0.621
Neutrophil-to-lymphocyte ratio, (>4.30 vs. ≤4.30)	0.170	1.186	0.804	1.747	0.390
LDH, (>165 vs. ≤ 165 IU/L)	0.063	1.065	0.746	1.521	0.729
ALP level, (>99 vs. ≤ 99 IU/L)	0.022	1.022	0.743	1.406	0.895
Lymphocyte absolute value, (≤0.93 vs. >0.93 ×10^9^/L)	0.054	1.055	0.682	1.631	0.810
D-Dimer (≥0.5 vs.<0.5 mg/L)	0.067	1.070	0.786	1.456	0.669
Fibrinogen (>4.50 vs. ≤4.30 g/L)	0.105	1.111	0.786	1.570	0.551
Albumin (≤40 vs. >40 g/L)	0.021	1.021	0.702	1.485	0.914
Brain metastasis (Yes vs. No)	0.177	1.193	0.863	1.649	0.284
Bone metastasis (Yes vs. No)	0.003	1.003	0.703	1.432	0.985
T stage (T_1-2_ vs, T_3-4_)	0.029	1.030	0.723	1.467	0.871

CI, confidence interval.

### Prognostic Model for OS

**Figure 1:**
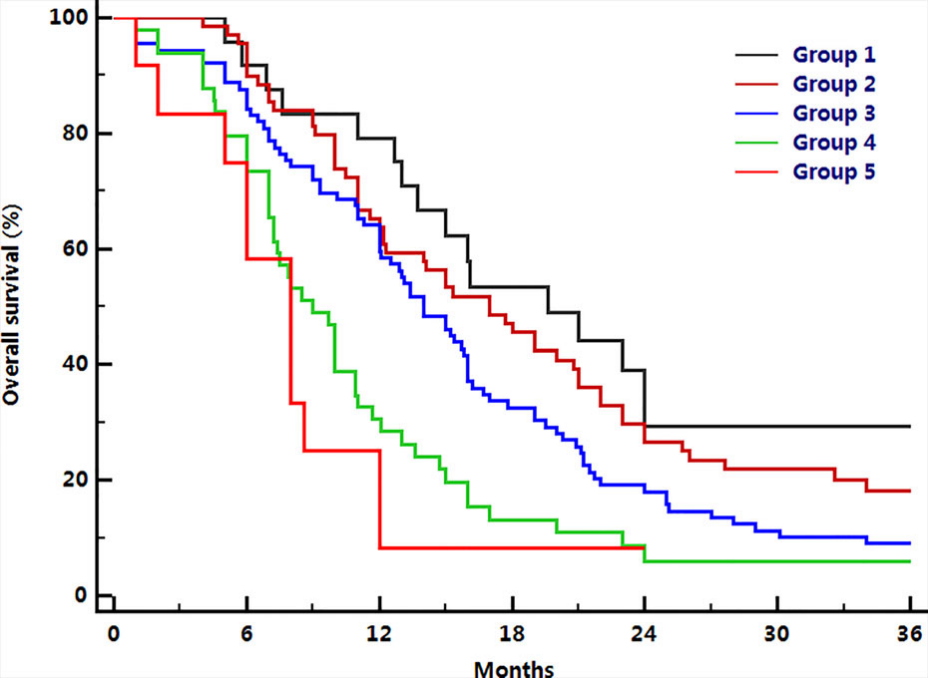
Comparison of overall survival among six groups.

A prognostic model was established based on 6 independent risk factors (being male, KPS score < 80, a number of chemotherapy cycles < 4, Hb level ≤ 120g/L, neutrophil count >5.8 ×10^9^/L, and PLT count >220 ×10^9^/L). Risk groups were defined by the number of presenting risk factors (0, 1, 2, 3, 4, 5, or 6). These 6 parameters were scored as “0” if there were no risk factors and ‘‘+1’’ if there was an additional risk factor. Therefore, a total prognostic score for each patient was calculated ranging from 0 to 6. All patients had at least one of these six risk factors, 24 patients had only one risk factor (group 1), 69 patients had two risk factors (group 2), 89 patients had three risk factors (group 3), 49 patients had four risk factors (group 4), 12 patients had five risk factors (group 5), and no patient had all six risk factors. The median OS time for these five groups were 19.6 (95% CI: 12.2–27.0), 17.0 (95% CI: 12.8–21.2), 14.0 (95% CI: 11.7–16.3), 9.0 (95% CI: 7.2–10.8), and 8.0 (95% CI: 5.9–10.1) months respectively (*P <*0.001; **Figure 1**). Group comparison analysis revealed no significant differences in OS between group 1 and group 2 (*P*=0.124), and between group 4 and group 5 (*P*= 0.334). Significant differences were observed among other groups (*P <*0. 05).

**Figure 2:**
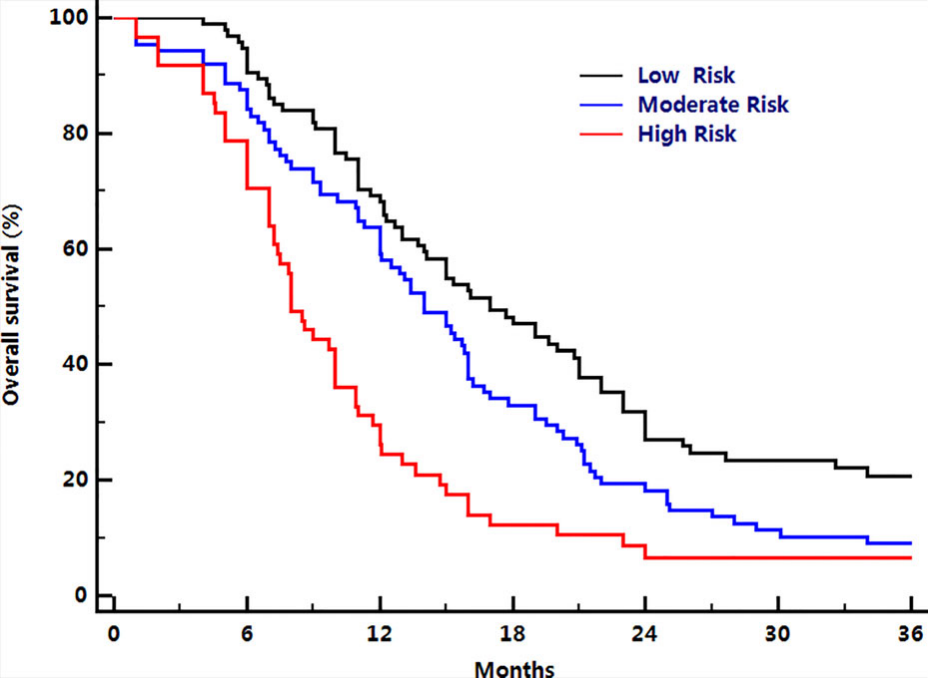
Comparison of overall survival among three different risk groups.

The number of patients was small in group 1, only 24 cases, and there was no significant difference in the overall survival outcomes between group 1 and group 2. Thus, group 1 and group 2 were merged into the same risk group. Based on the same consideration, group 4 and group 5 were also merged into the same risk group. Therefore, all patients were further assigned to one of three risk groups based on the number of presenting risk factors: those having ≤ 2 risk factors were scored as the low-risk group, those with 3 risk factors were scored as the moderate-risk group, and those with ≥ 4 risk factors were scored as the high-risk group. The median OS rates were 17.0 (95% CI: 13.3–20.7) months for the low-risk group, 14.0 (95% CI: 11.7–16.3) months for the moderate-risk group, and 8.0 (95% CI: 6.8–9.2) months for the high-risk group. The 1-year OS rates for low-risk, moderate-risk, and high-risk group were 67.7%, 59.6%, and 26.2%; 2-year OS rates were 32.1%, 18.0%, and 7.9%; 3-year OS rates were 19.3%, 7.9%, and 0%, respectively (*P*<0.001, **Figure 2**).

Among these three risk groups, group comparison analysis revealed significant differences in OS time between any two groups (low-risk: moderate-risk group, P= 0.023; low-risk: high-risk group, P< 0.001; moderate-risk: high-risk group, P< 0.001). Herrell’s c-index was 0.672. When the whole group was subdivided into those with oligometastatic (who had <5 metastases) (P<0.001) and non-oligometastatic diseases (who had ≥5 metastases) (P = 0.026), overall survival time among these three risk groups remained significant.

## Discussion

Around 60% of patients initially diagnosed with NSCLC have distant metastases (1). Recently, there has been increasing evidence demonstrating that a subset of patients with metastatic diseases could benefit from radiation therapy to the primary tumor with concurrent chemotherapy. However, not all patients could benefit from this treatment modality, and there is no consensus on the use of concurrent chemotherapy and radiotherapy for NSCLC (23). Therefore, we collected data from two prospective studies to identify patients with metastatic diseases who may benefit from chemotherapy administered concurrently with radiation therapy to the primary tumor. In this research, we developed a practical prognostic model based on laboratory and clinical parameters and demonstrated its predictive effect on the overall survival of metastatic NSCLC patients treated with chemotherapy with concurrent radiation to the primary tumor.

In this study, multivariate analysis showed that 6 factors have an effect on overall survival including two clinical parameters (sex, KPS score), one treatment-related parameter (the number of chemotherapy cycles), and three hematological parameters (Hb level, neutrophil, and PLT count).

As inflammation plays an important role in the pathogenesis and tumor progression in patients with NSCLC (29), hematological markers of systemic inflammation could be considered as potential prognostic factors for overall survival. Additionally, neutrophils and lymphocytes appear to be the main candidates for this role. Consistent with the result of a previous study (26), neutrophil count was related to overall survival in this study. It has been shown that tumor cells aggregate with PLTs, evade recognition by the immune system, adhere to distant vascular endothelial cells, and continue to metastasize, invade, and grow through the blood circulation (30). PLT counts in lung cancer patients are significantly increased compared with the healthy population, and high platelet count indicates poor prognosis for NSCLC patients (30, 31). Hemoglobin is an important biomarker for anemia. Cancer-related anemia is a multifactorial issue, which is associated with the nutritional, metabolic, and immune components of cancer, as well as the progression and severity of cancer (32). Hb level is a significant predictor of survival outcomes in patients with NSCLC (33, 34). Consistent with the result of previous publications, we also found neutrophil count, PLT count, and Hb levels were independent prognostic factors for OS in metastatic NSCLC patients treated with concurrent chemoradiotherapy.

According to ASCO guidelines, 4–6 chemotherapy cycles were recommended for stage IV NSCLC (35). We found that when patients were grouped according to the number of chemotherapy cycles, the overall survival time of patients receiving ≥4 cycles of chemotherapy was prolonged. In this study, multivariate analysis showed that the number of cycles of chemotherapy had a statistically significant effect on OS. Consistent with the result of previous publications, we also found that good performance status and female sex were associated with better OS (24, 25, 27, 36, 37).

The prognosis of metastatic NSCLC can be extremely different because of its heterogeneous characteristics, so it is necessary to establish a well-defined risk scoring system to predict the survival of metastatic NSCLC. Ulas et al. established a laboratory prognostic index (LPI) in advanced NSCLC patients based on hematologic and biochemical parameters. From their result, LPI combined with clinical parameters may help formulate individualized treatment plans and predict survival rates (25). Gagnon et al. developed a Montreal prognostic score based on LDH, albumin, CRP, and neutrophil lymphocyte ratio in incurable lung cancer patients. Montreal prognostic score divided patients into three distinct groups: the median OS times were 2.5, 8.2, and 18.2 months, respectively (log-rank, *P <*0.001) (38).

The risk of death is highly variable because of the interactions between clinical characteristics and treatment. However, there was no risk scoring system to predict the survival of metastatic NSCLC patients treated with chemotherapy with concurrent radiation to the primary tumor. This study classified patients into low-risk, moderate-risk, and high-risk groups based on six independent prognostic factors.

Previous publications showed that chemotherapy alone produces median overall survival time, and 1- and 2-year OS rates were approximately 8.0 months, 30.0%, and 10.0%, respectively for metastatic NSCLC (13, 39). In this study, the median OS were 17.0 months for the low-risk group, 14.0 months for the moderate-risk group, and 8.0 months for the high-risk group; the 1-year OS rates for the low-risk, moderate-risk, and high-risk groups were 67.7%, 59.6%, and 26.2%; 2-year OS rates were 32.1%, 18.0%, and 7.9%; 3-year OS rates were 19.3%, 7.9%, and 0% respectively. Our findings indicated that the combination of systemic chemotherapy and concurrent radiotherapy to the primary thoracic tumor could further improve survival for low-risk and moderate-risk patients. For high-risk patients, the addition of radiotherapy resulted in no improvement in survival compared with chemotherapy alone (13, 39, 40). Based on current data, we suggest risk-adapted therapy for metastatic NSCLC: low-risk and moderate-risk patients may benefit from radiotherapy to the primary tumor with concurrent chemotherapy, and high-risk patients might be treated with chemotherapy alone.

Literature data supported the role of local treatment in oligometastatic NSCLC disease (20, 22). However, there was no uniform definition of oligometastatic disease in NSCLC (16, 18, 20, 22, 41–43). According to our institutional data and other reports, nearly 90% of stage IV NSCLC patients have metastases confined to one to three organs (12, 14, 44). In this study, all patients had metastases confined to three or fewer organs (regardless of the number of metastatic lesions in each organ). In 2019, the European consensus group proposed a provisional definition of oligometastatic NSCLC as follows: maximum of five metastases and three organs (45). We found that the number of metastatic lesions were associated with OS in univariate analysis. When the entire group was divided according to the total number of metastases (< 5 metastases vs. ≥5 metastases), the prognostic model retained significance for predicting OS. Thus, we propose that this model can be applied to patients with oligometastatic (< 5 metastases) or non-oligometastatic (≥5 metastases) diseases.

We acknowledge several limitations in the current study. Pharmacotherapy has been the main treatment for metastatic NSCLC and still plays an irreplaceable role. In recent years, molecular targeted therapy and immunotherapy have yielded good survival outcomes in patients with metastatic NSCLC (46, 47). For metastatic NSCLC patients, thoracic radiation plus molecular targeted therapy or immunotherapy may produce better survival outcomes as compared with molecular targeted therapy or immunotherapy alone (19, 48, 49). Since none of the patients in this study have received molecular targeted therapy or immunotherapy, we cannot comment on whether the current predictive model is applicable for metastatic NSCLC patients treated with thoracic radiation in combination with targeted therapy or immunotherapy. Thus, it is necessary to further investigate a prognostic model for metastatic NSCLC with thoracic radiotherapy combined with targeted therapy or immunotherapy. Moreover, we did not directly compare survival outcomes between chemotherapy alone and chemotherapy with concurrent radiotherapy to the primary tumor in different risk subgroups. Therefore, we suggest further investigation on the efficacy of concurrent radiotherapy to the primary tumor in different subgroups of metastatic NSCLC treated with chemotherapy.

## Conclusions

Metastatic NSCLC patients treated with chemotherapy in combination with thoracic radiation were classified as low-risk, moderate-risk, or high-risk group using six independent prognostic factors (sex, KPS score, number of chemotherapy cycles, Hb level, neutrophil, and PLT count). Risk-adapted therapy of radiation to the primary tumor based on systemic chemotherapy for the low-risk or moderate-risk and chemotherapy alone for the high-risk group may be the appropriate treatment. The value of concurrent radiation for metastatic NSCLC patient needs to be further investigated in different risk subgroups, and additional studies are necessary to establish a predictive model for metastatic NSCLC treated with thoracic radiation in combination with targeted therapy or immunotherapy.

## Data Availability Statement

The original contributions presented in the study are included in the article. Further inquiries can be directed to the corresponding author.

## Ethics Statement

This study was reviewed by the ethical review boards in China (Ethics Committee of Guizhou Cancer Hospital, GuiYang, China). The patients/participants provided their written informed consent to participate in this study.

## Author Contributions

S-FS and BL designed the study. L-FL, Q-SL, Y-XH, W-GY, X-XC, ZM, W-WO, Y-CG, and CH collected the data. L-FL and Q-SL drafted and prepared the manuscript. All authors contributed to the article and approved the submitted version.

## Funding

This work was supported by grants from Guizhou Provincial Education Office, China [grant number KY (2016) 032] and Guizhou Province’s Science and Technology Major Project, China [grant number Qian-J Zhong (2015) 2003]. The funders were not involved in research design, information collection and analysis, decision to publish, or writing the manuscripts.

## Conflict of Interest

The authors declare that the research was conducted in the absence of any commercial or financial relationships that could be construed as a potential conflict of interest.
